# The Endogenous Cannabinoid and the Nitricoxidergic Systems in the Modulation of Stress Responses

**DOI:** 10.3390/ijms24032886

**Published:** 2023-02-02

**Authors:** Hristina Nocheva, Nikolay S. Krastev, Dimo S. Krastev, Milka Mileva

**Affiliations:** 1 Department of Physiology and Pathophysiology, Faculty of Medicine, Medical University, 1403 Sofia, Bulgaria; 2 Department of Anatomy, Faculty of Medicine, Medical University, 1606 Sofia, Bulgaria; 3 College of Medicine “Yordanka Filaretova”, Medical University, 1606 Sofia, Bulgaria; 4 Department of Anatomy and Physiology, South-West University “Neofit Rilski”, 2700 Blagoevgrad, Bulgaria; 5 The Stephan Angeloff Institute of Microbiology, Bulgarian Academy of Sciences, 1113 Sofia, Bulgaria

**Keywords:** anandamide (AEA), nitric oxide (NO), restraint stress (RS), antinociception, paw pressure test, hot plate test

## Abstract

The effects on stress-induced analgesia (SIA) from endogenous cannabinoid system (ECS) and nitric oxide (NO) interaction after 1 h of restraint stress were evaluated in male Wistar rats. The animals were subjected to 1 h of restraint and then injected with different combinations of cannabinoid receptor type 1 agonist anandamide (AEA) or antagonist AM251 along with an NO donor, NO precursor, or inhibitor of NO synthase. Nociception was evaluated using paw pressure (PP) or hot plate (HP) tests. AEA was administered immediately after the end of restraint-SIA (r-SIA). Administration of NO precursor reversed the pronociceptive effect of the CB1 agonist on r-SIA. Both the CB1 antagonist and the NOS inhibitor neutralized the pro-analgesic effect of L-arginine (L-arg). Administration of an NO donor, instead, increased r-SIA. Our experiments confirmed that the endogenous cannabinoid and the NO-ergic systems interact in the modulation of r-SIA. This interaction probably implies NO as a second messenger of the ECS.

## 1. Introduction

Disturbance of homeostasis in the human organism can be caused by many factors, both external and internal to the organism, and all of them usually lead to consequences that we generally define as stress. Susceptibility to stressors in different periods of development changes according to genetic predisposition. Changes occur in the body on many levels: morphological, physiological, emotional, etc. In the end, all these changes model the external manifestation of the behavioral reactions of the organism to the relevant stimulus with a single goal: adaptation to the changed conditions [1]. Adaptation processes to the dulling or strengthening of the perception to the stressogenic factor also occur at the biochemical level, which is predetermined by the structures of the synapses and the changes that occur during the aging process. They are manifested both at the level of cell membranes of the cytological objects that perceive the irritation, and in the changes of some of the receptors, because of their impaired biochemical activity. Regardless of the cause, stress affects all levels of the body’s vital functions and changes a large part of the adaptive functions, damaging the immune system, which affects the biological behavior controlled by the limbic system [2]. The limbic system is responsible for the behavioral reactions of the organism [3].

The axis on which the balance (homeostasis) of the human body depends includes various brain structures and the glands of the neuro-endocrine system [4], which includes the hypothalamus, pituitary gland, adrenal glands, and gonads. The “big boss” of this axis is undoubtedly the thyroid gland; it balances and regulates the unity of metabolic processes [5,6,7,8].

Stress induces functional and structural changes in the organism, triggering interactions among the central nervous, endocrine, and immune systems. Derangements in the control of such systems after short-lasting severe or mild but long-lasting stress can trigger several diseases, named “stress-induced diseases”: coronary heart disease, arterial hypertension, diabetes mellitus, peptic ulcer disease, Graves’ disease, malignancies, depression, reproductive disorders, etc. [9].

During stress, different physiological parameters change, e.g., pain perception, which is known to decrease stress-induced analgesia (SIA) [10].

Two components take part in SIA development: an opioid and a nonopioid component [11]. Nonopioid SIA includes several neurotransmitter systems, e.g., the adrenergic, the serotonergic, and the endogenous cannabinoidergic, all of which modulate pain perception and behavioral responses to stress [12,13].

The ECS includes two types of receptors (cannabinoid receptors type 1 and 2 (CB1 and CB2)) and their endogenous ligands (endocannabinoids, eCBs), e.g., arachidonoylethanolamide, better known as anandamide (AEA). The CB1 receptor is vastly distributed in the central and peripheral nervous system. eCBs are synthesized “on demand” by postsynaptic neurons, but their effects are presynaptically evident, which is known as retrograde signaling. CB1 activation decreases the release of several neurotransmitters from the presynaptic ends. Such an interaction with other neurotransmitters, as well as modulation of their activity, allows the ECS to fulfill its physiological effects on brain function and synaptic activity. The ECS also takes part in anatomical circles responsible for stress response and pain perception. Cannabinoids decrease nociception due to inhibition of ATP synthesis and G-protein activation [13,14,15,16,17,18].

The ECS, along with the opioidergic, adrenergic, and nitricoxidergic systems, was discovered to take part in the descending antinociceptive system [17,18].

NO derives from L-arginine (L-arg). Several specific forms of the enzyme NO synthase participate in its formation: neuronal and non-neuronal (endothelial and inducible) [19]. 

NO takes part in several physiological and pathophysiological processes. It is known to modulate both acute and chronic pain perception at central and peripheral levels, with its role being complex and somehow ambivalent. Even though the precise mechanisms of NO effects are not yet entirely understood, several possibilities have been proposed:

- After NO binds to its intracellular receptor, soluble guanylyl cyclase, its activation results in conversion of guanosine triphosphate to the second messenger cyclic guanosine monophosphate (cGMP); the NO–cGMP signaling pathway could be implicated in synaptic plasticity, e.g., central sensitization [20].
- NO diffusing out of the neuron acts as a neurotransmitter on nerve endings and astrocyte processes, enhancing the release of SP and, thus, contributing to the development of secondary hyperalgesia [21].
- At the spinal level, NO participates in descending facilitation, weakening the influence of descending inhibition upon dorsal horn neurons, at least partially, by interfering with GABAergic and glycinergic inhibitory tone upon projection neurons [22,23].

The abovementioned examples of NO effects point to interactions with other mediators/modulators. Some of the effects of eCBs have also been documented to be mediated by NO, by inhibiting the release of other mediators [24]. During stress, NO levels are known to increase; NO has been proposed as the retrograde messenger engaged in the regulation of both pre- and postsynaptic mechanisms involved in synaptic plasticity [19,25].

Uncovering the mechanisms underlying the body’s stress response can provide guidance for controlling the stress reaction and its adverse consequences. At the same time, pain itself represents a stressful event with serious consequences for the individual. Both the endocannabinoid and the nitricoxidergic systems, on the other hand, have been associated with stress and nociception/analgesia.

The objective of this study was to evaluate whether the exogenous manipulation of the ECS and the NOergic system can affect the stress response, as evaluated by the changes in pain perception, and whether this potential interaction increases or decreases stress-induced analgesia, a well-known phenomenon that develops after stress exposure. 

## 2. Results

### 2.1. Effects of AEA and L-Arg on r-SIA

**Figure 1:**
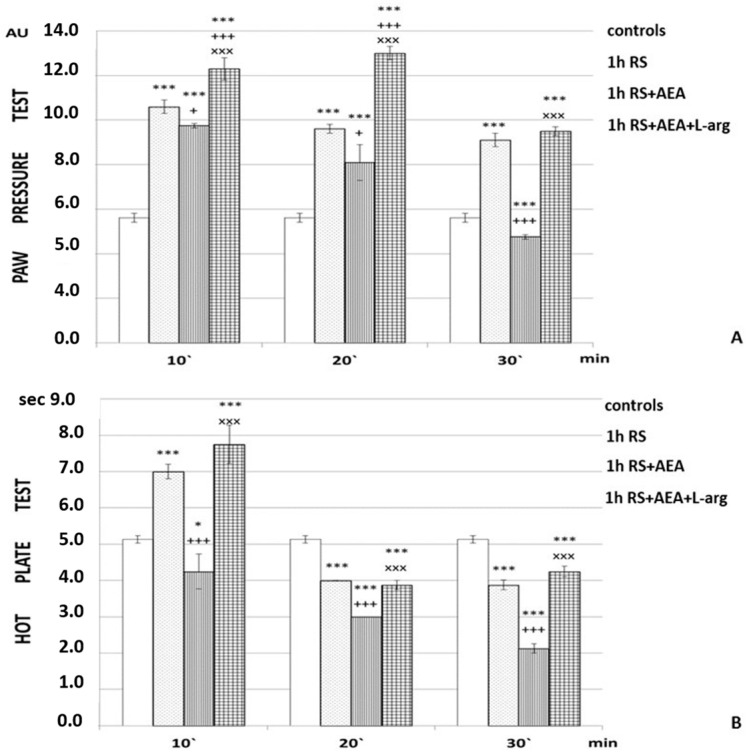
Effects of AEA and L-arg on r-SIA estimated by (**A**) PP test and (**B**) HP test after 1 h of restraint (1 h RS) in rats. The mean values ± SEM are presented. *** *p* < 0.001, * *p* < 0.05 vs. control; ^+++^ *p* < 0.001, ^+^ *p* < 0.05 vs. RS; ^xxx^ *p* < 0.001 vs. RS + AEA; ^+++^ *p* < 0.001 (F = 37.8692 at the 10th min; F = 302.4439 at the 20th min); 1 h RS + AEA group (F = 161.55249 at the 10th min; F = 609.1875 at the 20th min).

It was determined that 1 h of RS increased the PPT for the entire time of the experiment (*p* < 0.001; F = 779.67482 at the 10th min; F = 432.31579 at the 20th min; F = 376.2809 at the 30th min), as well as the HPL at the 10th min compared to the control (Figure 1).

Administration of the combination AEA + L-arg at the end of 1 h of RS increased the PPT at the 10th and 20th min (*p* < 0.001) compared to animals from the 1 h RS (F = 37.8692 at the 10th min; F = 302.4439 at the 20th min) and 1 h RS + AEA groups (F = 161.55249 at the 10th min; F = 609.1875 at the 20th min), with higher values at the 20th min. At the 30th min, PPT results were comparable to the 1 h RS experimental group (Figure 1A). HPL results were comparable to the 1 h RS group throughout the experiment (Figure 1B).

### 2.2. Effects of AEA and L-Arg with AM251 Pretreatment

In another experimental trial, a CB1 receptor antagonist was administered in order to evaluate the relevancy of cannabinoids for the analgesic effect described.

**Figure 2:**
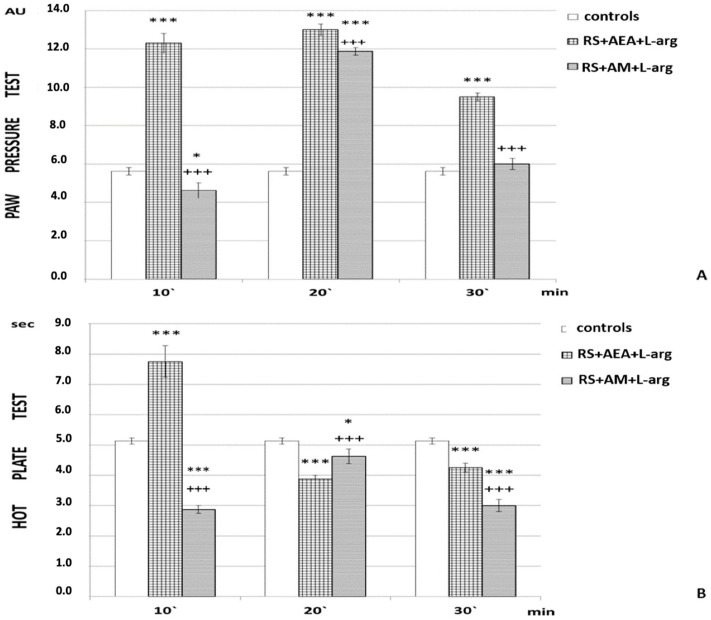
Effects of AEA and L-arg with AM251 pretreatment on r-SIA estimated by (**A**) PP and (**B**) HP test after 1 h of restraint (1 h RS) in rats. The mean values ± SEM are presented. *** *p* < 0.001, * *p* < 0.05 vs. control; ^+++^ *p* < 0.001 vs. 1 h RS + AEA + L-arg. *** *p* < 0.001 (F = 1226.68966 at the 10th min; F = 57.14286 at the 20th min; F = 402.2069 at the 30th min).

AM251 pretreatment (1 h RS + AM + L-arg) decreased the PPT of the experimental animals compared to animals after 1 h RS + AEA + L-arg (*p* < 0.001; F = 1226.68966 at the 10th min; F = 57.14286 at the 20th min; F = 402.2069 at the 30th min) for the whole time of the experiment (Figure 2). Pain thresholds were comparable to the control at the 10th and 30th min of the experiment, while, at the 20th min, a transient increase was detected (Figure 2A).

On HP evaluation, a tendency toward hyperalgesia was observed at the 10th and 30th min. At the 20th min, a transient elongation of HPL was also observed (Figure 2B).

### 2.3. Role of NO

The role of NO was estimated by inhibition of NO synthase. L-NAME injection (1 h RS + AEA + L-NAME) decreased the PPT (*p* < 0.001) throughout the experiment compared to the 1 h RS + AEA + L-arg group. It is worth mentioning that, at the 20th min, a transient increase in PPT was again observed (*p* < 0.001; F = 1001.73346 at the 10th min; F = 232.52248 at the 20th min; F = 536.92814 at the 30th min).

**Figure 3:**
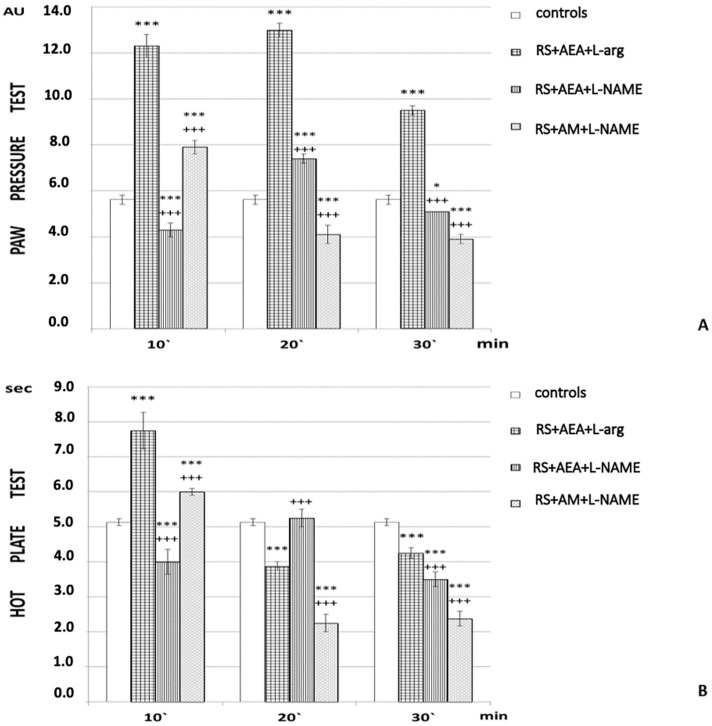
Effects of AEA and L-arg with L-NAME AM251 + L-NAME pretreatment estimated by (**A**) PP and (**B**) HP tests after 1 h of restraint (1 h RS) in rats. The mean values ± SEM are presented. *** *p* < 0.001, * *p* < 0.05 vs. control; ^+++^ *p* < 0.001 vs. 1 h RS + AEA + L-arg; *p* < 0.001 (F = 1001.73346 at the 10th min; F = 232.52248 at the 20th min; F = 536.92814 at the 30th min).

An inverted U-shaped curve was also observed for HPL of 1 h RS + AEA + L-NAME animals; a tendency toward hyperalgesia was detected at the 10th and 30th min of the experiment (Figure 3B).

Pretreatment with both AM251 and L-NAME (1 h RS + AM + L-NAME) abolished analgesia registered following the combination AEA + L-arg after the 20th min, and a tendency toward hyperalgesia was evaluated by both PP and HP tests (Figure 3A,B).

### 2.4. Role of NO-Donor SIN-1

An additional experimental trial was performed with NO-donor SIN-1 application after L-NAME or after both AM251 and L-NAME.

**Figure 4:**
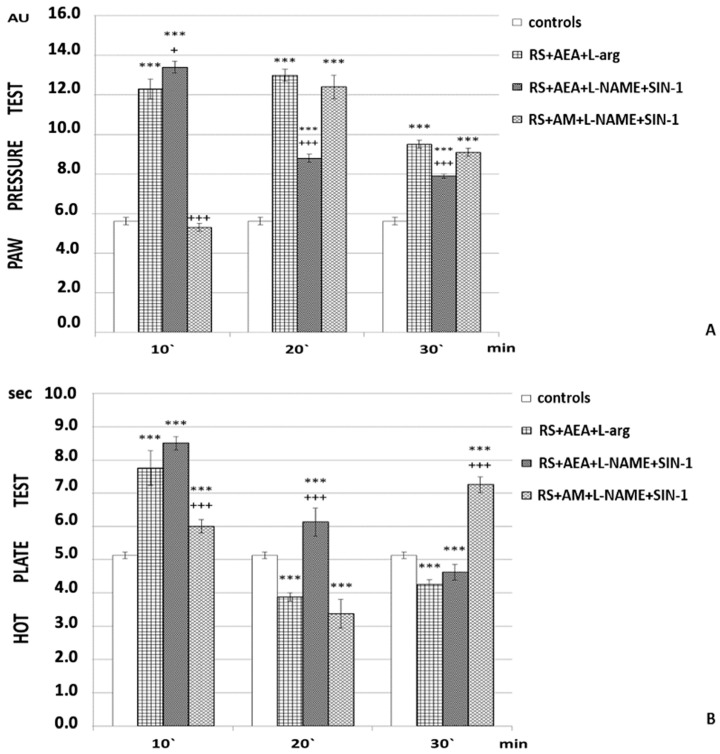
Effects of SIN-1 with L-NAME alone or AM251 + L-NAME pretreatment estimated by (**A**) PP and (**B**) HP tests after 1 h of restraint (1 h RS) in rats. The mean values ± SEM are presented. *** *p* < 0.001 vs. control; ^+++^ *p* < 0.001, ^+^ *p* < 0.05 vs. 1 h RS + AEA + L-arg.

In the 1 h RS + AEA + L-NAME + SIN-1 animals, progressively decreasing PPT results were estimated compared to the 1 h RS + AEA group. Yet, PP thresholds were higher than the control (Figure 4A).

HPL results also progressively decreased; nevertheless, they were longer than those of the 1 h RS + AEA groups until the 30th min, at which point they became comparable (Figure 4B).

SIN-1 application after both AM251 and L-NAME (1 h RS + AM + L-NAME + SIN-1) led to PPT results comparable to those of animals after 1 h RS + AEA + L-arg from the 20th min until the end of the experiment (Figure 4A). HPL results were shorter than the 1 h RS + AEA + L-arg group at the 10th min, comparable at the 20th min, and longer at the 30th min of the experiment (Figure 4B).

## 3. Discussion

In the present work, we investigated the interaction between the ECS and NO after 1 h of restraint and its effect on the degree of r-SIA. The obtained results showed that administration of an NO precursor neutralized the pro-nociceptive effect of AEA on r-SIA (Figure 1A). Under conditions of stress, all systems in the body go into counteraction mode, because of adaptation to new conditions [26]. Changes occur in all adaptation markers. SIA is a very extensively explored phenomenon; the decrease in pain perception during acute stress is a necessary change in sensory awareness, aimed at preserving the ability to focus on potentially life-threatening factors. Since stress itself is potentially dangerous for the body, due to the possibility for the development stress-induced pathology, approaches to decrease the level of stress could be useful in the prevention of the negative consequences from stress exposure. Different systems are known to interact in the pathogenesis of the body’s stress response. Discovery of the underlying mechanisms could give new directions for preventing and counteracting stress-induced pathology. Both the endogenous cannabinoid and the nitric oxide systems take part in SIA development.

Historically, more than 5000 years ago, cannabis was among the first plants used as medicine, in religious rituals, and for recreational purposes. It later became known that cannabis contains more than 66 components, so-called cannabinoids, which can interact with endogenous cannabinoid systems in the human body [27,28].

Nevertheless, the beneficial health effects of cannabinoids, for the most part, remain empirical and anecdotal. Since the discovery of the endocannabinoid system, consisting of cannabinoid receptors, endogenous ligands, and biosynthetic and metabolizing enzymes, the specificity of the inflammatory response has largely been elucidated.

Over the past several decades, significant progress has been made in understanding the receptors and enzyme systems that make up the endocannabinoid system. The effects observed during its modulation or dysregulation can be numerous and varied.

In practice, under stress, immune cells receive danger signals associated with pathogenic damage-associated pattern recognition receptors [29]. The complex relationship between the endocannabinoid system and the immune system involves multiple cellular signaling mechanisms regulating various physiological and neurotransmission pathways in the mammalian brain, including activation of the dopaminergic system [28].

It should be emphasized that the integration and function of the various components of the endocannabinoid system are complex, and that its modulation affects various physiological processes that continue to be the subject of serious scientific research [30,31]. Therefore, immune cells are involved in the regulation of endocannabinoid homeostasis; in turn, the endocannabinoid system modulates local inflammatory responses. This necessitates the study of new approaches in the development of therapeutic strategies for the control of chronic inflammatory diseases [32].

The ubiquitous distribution of the cannabinoid receptors presumes the ECS’s participation in different processes of physiological and pathological significance. In this relationship, the interaction between the ECS and other systems is interesting because of the possible effect on SIA. In our experiments, SIA was taken as an indirect indicator for a stress reaction, and changes in pain perception reflected the impact of the cannabinoid receptor activity on the level of stress. Furthermore, since some investigations highlighted similarities in the behavior of animals subjected to social and restraint stress procedures [33,34], we chose the restraint stress model for our study. 

Given the dual effect of NO on nociception [19], the authors were “open” to the dual possibility of both a potentiation of and a decrease in r-SIA. The results obtained were not surprising but birthed some interesting questions about the interactions between cannabinoids and the nitricoxidergic system after restraint stress.

As it turns out, administration of an NO precursor neutralized the pro-nociceptive effect of AEA on r-SIA. The two systems obviously interacted together; however, it is not clear how they are connected.

Antagonization of the CB1 receptor or inhibition of NO synthesis decreased PPT and shortened HPL, with L-NAME seeming to exert a more prominent effect than AM251. Yet, an interesting shift in the effect was observed at the 20th min, i.e., a transient increase in PPT and an elongation of HPL. 

An interaction between NO and cannabinoid receptor agonists at the level of potassium and calcium channels could be a possible explanation for the results described [35,36]. It is also possible that NO itself represents a second messenger for the ECS, and that activation of CB1 receptors leads to activation of NO synthase [24]. The increase in r-SIA following SIN-1 application after both AM251 and L-NAME could be considered a confirmation of such a theory; introduction of an NO donor increased r-SIA to the levels observed after AEA and L-arg, even with the effect becoming visible later. Another possibility is that ECS and NO interacted through engaging the same second messengers [37].

U-shaped curves were repeatedly observed during the experiments and represent another interesting finding. Obviously, the interaction between the ECS and the NOergic system is not always unidirectional. A possible explanation could be that some foreign factor superposed with the interaction between the ECS and the NOergic system at the 20th min of the experiments, since it is known that cannabinoids modulate the responses of other mediatory systems. Additional experiments could be useful to clarify the interactions with other mediatory systems in the modulation of SIA. 

## 4. Materials and Methods

### 4.1. Experimental Animals 

Experiments were carried out on male Wistar rats (180–200 g) kept under normal conditions at ambient room temperature (22 °C). The animals were divided into seven experimental groups, each including 8–10 animals, and a control group (n = 10). All experimental procedures were carried out between 10:00 a.m. and 1:00 p.m. after approval from the Research Ethics Commission of the Medical University of Sofia. The experimental protocols were approved by the Bulgarian Food Safety Agency (BFSA), Permission No. 288/22.10.2020.

The experimental animals were subdivided into seven groups, along with some control groups:

Group 1—animals after 1 h of restraint stress injected with AEA,
Group 2—animals after 1 h of restraint stress injected with AEA and L-arg,
Group 3—animals after 1 h of restraint stress injected with AM and L-arg,
Group 4—animals after 1 h of restraint stress injected with AEA and L-NAME,
Group 5—animals after 1 h of restraint stress injected with AM and L-NAME,
Group 6—animals after 1 h of restraint stress injected with AEA, L-NAME, and SIN-1,
Group 7—animals after 1 h of restraint stress injected with AM, L-NAME, and SIN-1.

In addition to the groups described above, considered “experimental”, we also had several groups considered “controls” in which PPT and HPL were evaluated: animals without any stress; animals injected with saline (the “controls” in Figure 1, Figure 2, Figure 3 and Figure 4); animals injected with DMSO; animals subjected to 1 h of restraint without substances administrated (1 h RS in Figure 1, Figure 2, Figure 3 and Figure 4).

### 4.2. Acute Model of Restraint Stress (1 h RS)

The animals were placed for 1 h in plastic tubes with adjustable plaster tape on the outside to prevent moving. Holes were left for breathing. No food and water were available during the time of restraint. 

### 4.3. Drugs and Treatment 

All drugs were obtained from Sigma Aldrich (Merck, Sofia, Bulgaria) and administered intraperitoneally (*i.p*). The CB1 receptor agonist anandamide (AEA, at a dose of 1 mg/kg BWT) and the CB1 receptor antagonist AM251 (at a dose of 1.25 mg/kg BWT) dissolved in DMSO were injected immediately after the end of stress. L-arginine (L-arg, an NO precursor) was applied at a dose of 1 mg/kg BWT; the inhibitor of NO synthase, L-NAME, was applied at a dose of 10 mg/kg BWT; a combination of L-NAME followed by the NO donor SIN-1 (0.2 mg/kg BWT) was also applied.

The substances were not injected simultaneously; the CB1 agonist (AEA) was administered first, immediately after the end of stress, in groups 2, 4, and 6; in groups 3, 5, and 7, the antagonist (AM251) was instead the first to be administered at the end of the stress. 

L-arg (in groups 2 and 3) or L-NAME (in groups 4, 5, 6, and 7) were administered after the CB1 agonist/antagonist (in a different syringe); additionally, SIN-1 was administered separately in groups 6 and 7 after L-NAME.

### 4.4. Paw Pressure Test (Randall–Selitto Test)

Changes in the mechanical nociceptive thresholds (PPT) of experimental animals were measured using an Ugo Basile analgesiometer [38]. Pressure was applied to the hind-paw, and the value (g) required to elicit a nociceptive response (such as a squeak or struggle) was taken as the mechanical nociceptive threshold. A cutoff value of 500 g was used to prevent damage of the paw.

### 4.5. Hot Plate Test 

The HP latency (HPL) of the response to pain was measured from the moment an animal was placed on a metal plate (heated to 55 ± 0.5 °C) until the first signs of pain (paw licking or jumping). A cutoff time of 30 s was observed. HPL values were estimated using Ugo Basile hot/cold plate.

### 4.6. Data Analysis 

The results were statistically assessed using one-way analysis of variance followed by the Newman–Keuls post hoc comparison test. Values are represented as the mean ± SEM. A *p*-value < 0.05 was considered to indicate statistical significance.

## 5. Conclusions

AEA administration immediately at the end of stress decreased the r-SIA. Interaction of the ECS and the nitricoxidergic system contributed to r-SIA modulation. Administration of an NO precursor reversed the pro-nociceptive effect of the CB1 agonist on r-SIA. Both the CB1 antagonist and the NOS inhibitor neutralized the pro-analgesic effect of L-arg. Administration of an NO donor, instead, increased r-SIA.

These interactions probably imply NO as a second messenger of the ECS.

## Data Availability

All experimental protocols and data are stored in the archives of the Department of Physiology and Pathophysiology, Faculty of Medicine, Medical University of Sofia, 1403 Sofia, 2, Zdrave Str., Bulgaria.
